# Immune Monitoring for Advanced Cell Therapy Trials in Transplantation: Which Assays and When?

**DOI:** 10.3389/fimmu.2021.664244

**Published:** 2021-03-25

**Authors:** Helen L. Stark, Hayson C. Wang, Jasmina Kuburic, Alaa Alzhrani, Joanna Hester, Fadi Issa

**Affiliations:** ^1^ Transplantation Research and Immunology Group, Nuffield Department of Surgical Sciences, University of Oxford, Oxford, United Kingdom; ^2^ Division of Plastic Surgery, Peking Union Medical College Hospital, Peking Union Medical College and Chinese Academy of Medical Sciences, Beijing, China

**Keywords:** immune monitoring, cell therapy, transplantation, regulatory T cell, mesenchymal stromal cell

## Abstract

A number of immune regulatory cellular therapies, including regulatory T cells and mesenchymal stromal cells, have emerged as novel alternative therapies for the control of transplant alloresponses. Clinical studies have demonstrated their feasibility and safety, however developing our understanding of the impact of cellular therapeutics *in vivo* requires advanced immune monitoring strategies. To accurately monitor the immune response, a combination of complementary methods is required to measure the cellular and molecular phenotype as well as the function of cells involved. In this review we focus on the current immune monitoring strategies and discuss which methods may be utilized in the future.

## Introduction

The long-term treatment of transplant patients with immunosuppressive drugs is associated with significant side effects including life-threatening infections, cancer development, and direct drug toxicity (1–3). A number of immune regulatory cellular therapies including regulatory T cells (Tregs) and mesenchymal stromal cells (MSCs) have emerged as novel alternative therapies for the control of transplant alloresponses (4–6), with the possibility of reducing the morbidity associated with standard immunosuppression.

**Figure 1:**
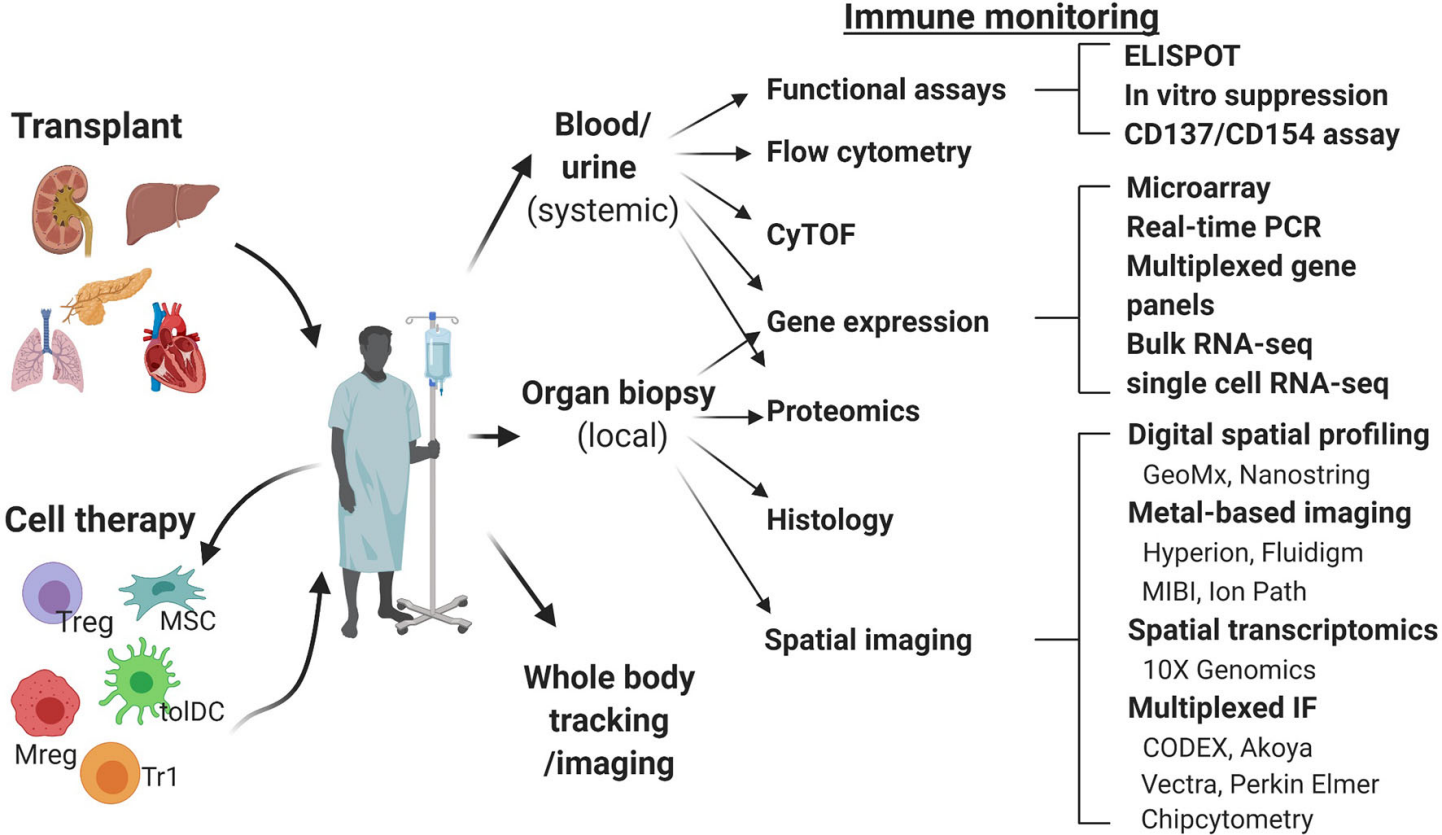
Schematic overview of the immune monitoring methods useful in cell therapy trials in transplantation. CODEX, co-detection by indexing; CyTOF, cytometry of the time of flight; IMC, imaging mass cytometry; MIBI, multiplexed ion beam imaging; Mreg, regulatory macrophage; MSC, mesenchymal stem cell; RT-qPCR, real time quantitative polymerase chain reaction; tolDC, tolerogenic dendritic cell; Treg, regulatory T cell; Tr1, type 1 regulatory T cell.

To date, clinical studies of advanced cellular therapies have focused on feasibility and safety. As the goal of these cellular therapies is to modify the immune response to transplantation, detailed immune monitoring in these trials is crucial. This immune monitoring facilitates a deeper understanding of the alloresponse, while providing crucial data on treatment effectiveness as well as the potential to identify new biomarkers or therapeutic targets. The immune response is a diverse and dynamic system that interacts temporospatially at many points, and it is therefore not possible (nor relevant) to monitor a single cell type in isolation. To accurately monitor the immune response, a combination of complementary methods is required to measure the cellular and molecular phenotype as well as the function of cells involved. In this review we will discuss the current methods of immune monitoring (**Figure 1**) and how they have the potential to become standard features of clinical trials in the future.

**Figure 2:**
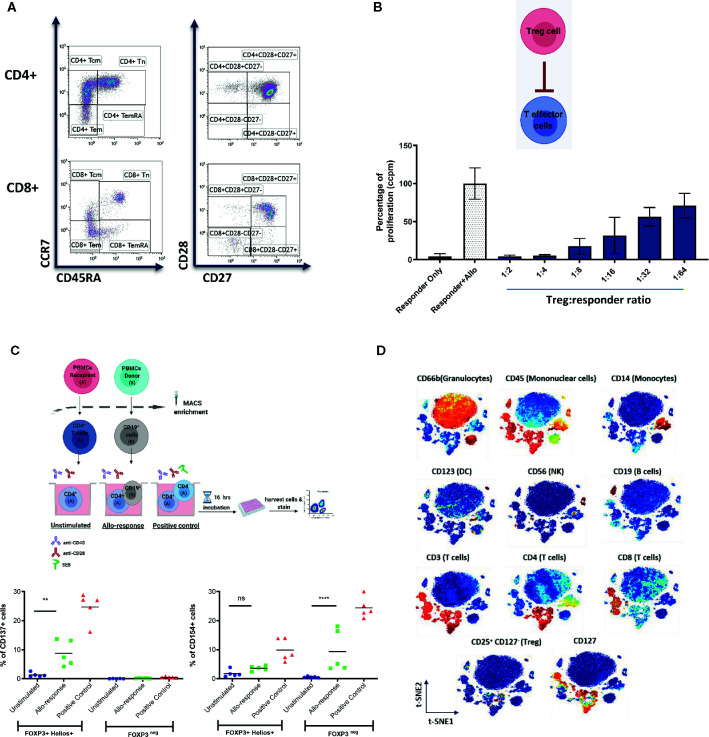
Representative examples of systemic immune monitoring techniques. **(A)** Flow cytometry can be used to measure the frequency of various immune cell populations in peripheral blood. **(B)** In vitro suppression assays can be used to assess the suppressive potential of Tregs (or other regulatory cells). Example of 3H-thymidine incorporation readout in a test with allogeneic stimulation. **(C)** (Top) Schematic of the experimental design of CD137/CD154 assay. (Bottom) Example of CD137 (left) and CD154 (right) expression on FOXP3^+^Helios^+^ (Tregs) and FOXP3^neg^ (conventional CD4+ T cells) cells. **(D)** Multiplexed CyTOF technology can be used for deep phenotyping analysis of leukocyte composition. An example of t-distributed Stochastic Neighbor Embedding (t-SNE) analysis of leukocyte clusters annotated based on the intensity of analyzed parameter is shown. ** = p value 0.0041, **** = p value < 0.0001, F test of variance. not significant (p >0.05).

**Figure 3:**
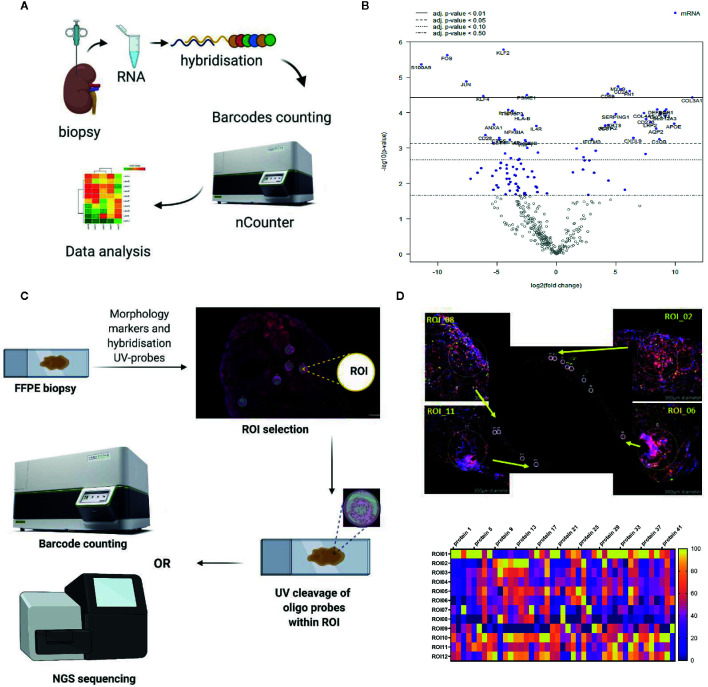
Representative examples of the *in situ* immune monitoring techniques. **(A)** Multiplexed gene expression using the nCounter, Nanostring. Schematic of the technique. **(B)** An example volcano plot obtained using Banff - Human Organ Transplantation (B-HOT) panel on the nCounter system, kidney biopsy versus background of PBMC. **(C)** Schematic of digital spatial profiling (DSP). **(D)** Example of kidney biopsy from renal transplant recipient analyzed using DSP (GeoMx, Nanostring). Morphology staining, selected ROIs and heat map analysis are shown. Example of geometric regions of interest (ROIs) and readout with panel of antibodies. FFPE, formalin fixed paraffin embedded; ROI, region of interest.

Currently, peripheral blood is the most studied material, due to the availability of well-developed techniques of analysis and ease of repeated sampling. However, data from pre-clinical models demonstrating homing of adoptively transferred human regulatory cells to the allograft and its importance for the induction of immune tolerance highlights the need for and value of allograft tissue analysis (7, 8). We will review here the methods most commonly used for systemic immune monitoring of blood, including flow cytometry, mass cytometry, functional assays and gene expression analysis (**Figure 2**) and discuss novel techniques of tissue biopsy analysis, including gene expression analysis and spatial biology methods (**Figure 3**).

## Flow Cytometry

Flow cytometry is well established as a technique for investigating the immune response. It provides rapid multi-parametric analysis of single cells in solution and is a powerful tool for immune monitoring as it can measure multiple parameters in parallel (example of data shown in **Figure 2A**) (9). Flow cytometers utilize lasers as light sources to produce both scattered and fluorescent light signals that are captured and converted into electronic signals (10). Cells are typically stained with fluorophore-conjugated antibodies or fluorescent dyes (10). This fluorescence is one of the limitations of the method, as the number of markers that can be analyzed concomitantly is limited by spectral overlap of the fluorophores used.

Whilst it is a commonly used method it is acknowledged that there can be significant variability in how samples are run and analyzed. This is of particular concern for clinical trials across different sites where it would be advantageous to be able to compare results. Efforts have been made to address this, Lee et al. published a consensus, the Minimum Information about a Flow Cytometry Experiment (MIFlowCyt) standard, detailing the minimum information that should be reported when publishing results of flow cytometry experiments (11), in order to aid both comparison and replication of results. Geissler et al. published the outcome of a TTS (The Transplant Society) symposium, where it was agreed it would be beneficial to establish consensus standard operating procedures (SOPs) for immune monitoring, the Virtual Global Transplantation Laboratory (vGTL) (12). To date, two SOPs, blood collection and PBMC isolation (13), and donor alloantigen specific IFNγ ELISpot (14) have been published. Expanding on this, Cossarizza et al. published invaluable guidelines for the use of flow cytometry in immunological studies, covering in detail the various techniques and applications of flow cytometry as well as how to analyze the results (15).

The ONE study was set up to study the immune-modulatory effect of a range of different regulatory immune cells in renal transplant patients (16). A key part of the setup was the development of a rigorous immune monitoring program, to profile the peripheral blood immune phenotype, using flow cytometry. Antibody panels were developed to profile T cell, B cell and dendritic cell (DC) subsets and their activation status (now available from Beckmann Coulter, DURAclone panels) (17). Streitz et al. reported on the results of this optimization process, including the strategy of local sample preparation using strict standard operating procedures (SOPs), followed by central analysis. They showed acceptable variability in results between multiple international sites. Using these standardized protocols provides results that can be compared between treatment groups and patients across multiple centers, vital for immune monitoring in clinical trials (17). The same panels have been incorporated by other cell therapy trials, like the TWO study (a randomized, phase II study investigating efficacy of regulatory T cells in kidney transplantation) and the Neptune study (a phase I study investigating mesenchymal stromal cells in renal transplantation).

The majority of trials looking at advanced cellular therapies in transplantation have used some form of flow cytometry analysis as part of their immune monitoring (16, 18–25). From the more basic measurement of numbers and proportions of different immune cell subsets, to following changes in the immune compartment over time. Below is a brief overview of how flow cytometry has been used for immune monitoring following cellular therapy.

### The ONE study

There were six different cell based medicinal products (CBMPs) trialed in the ONE study, including polyclonal Tregs (pTregs), donor alloreactive Tregs (darTregs), autologous tolerogenic dendritic cells (ATDC) and regulatory macrophages (Mregs). The immune monitoring methods detailed by Streitz et al. (17), were utilized to allow a metanalysis of the results. There was no difference in the CD4^+^CD25^high^CD127^low^Tregs at 60 weeks in those receiving CBMPs compared to standard care (16). However, there was evidence of significant decreases in Treg specific demethylated region (TSDR) demethylation in the standard care group. Furthermore, there was an increase in CD8^+^T_EMRA_ and CD8^+^CD57^+^ chronically active T cells in the standard care group. Both the CBMP group and standard care groups had more plasmacytoid dendritic cells at 60 weeks post-transplant than healthy age/sex matched controls. Interestingly there was a normalization of marginal zone-like B cell numbers and a significant reduction in CD14^high^CD16^+^ monocytes in those who received a CBMP. Taken together this suggested that those who received CBMPs had restoration of an immune phenotype more similar to the healthy controls than those receiving standard of care (16).

### Regulatory T cells

In 2014 the TRACT (Treg adoptive cell therapy) trial was commenced, a phase I trial to test safety and to trial escalating doses of autologous polyclonal Treg therapy in kidney transplantation (18). Flow cytometric analysis was the main method of immune monitoring of patients in this trial. Of note, patients had induction therapy with alemtuzumab, resulting in a significant decrease in T cells as well as B cells, NK cells and CD14^+^ monocytes in the first month post-transplant (18). By day 90 numbers of the majority of these cells had recovered, however numbers of CD4^+^ and CD8^+^ T cells remained low (18). Interestingly, the authors observed an increase in Tregs that remained stable at 1 year (18).

Similarly, Todo et al. used flow cytometry as part of the immune monitoring strategy for patients who underwent liver transplantation combined with Treg therapy (19). They also noted a trend to increased Treg (CD4^+^CD25^+^CTLA4^+^/CD4^+^CD25^+^Foxp3^+^) numbers post transplantation, however, they were unable to demonstrate a significant difference due to variation between recipients (19). Sánchez-Fueyo et al. used ONE study panels for immune monitoring of autologous polyclonal Treg therapy in liver transplantation. They did not find any significant changes in immune cell subsets post Treg therapy (25).

### Mesenchymal Stromal Cells

The Neptune study, a phase I study of allogeneic mesenchymal stromal cells (MSCs) in kidney transplant recipients (22), utilized the same flow cytometry protocols and panels as the ONE study for immune monitoring of participants, allowing detailed monitoring of the changes in leukocyte subsets post cell therapy (22). Authors found a decrease in CD19^+^ B cells, CD56^+^ NK cells, CD8^+^ T cells, CD4^+^ T cells and Tregs post induction immunotherapy, as expected (22). The B cells and NK cells began to recover from week 25 post induction (22). Although showing signs of repopulation, T cell numbers had not returned to baseline by 12 months (22). However, CD4^+^ T cells showed a significant increase in number after two MSC infusions (22). Later timepoint data are awaited from this trial.

Perico et al. used flow cytometry for immune monitoring of a phase I trial of autologous MSCs in renal transplantation (21). Initially they demonstrated a profound depletion of CD8^+^ and CD4^+^ T cells (21). The CD8^+^ T cell numbers recovered by day 360, however CD4^+^ T cells never regained pretransplant levels (both control and MSC group) (21). The MSC group had fewer CD8^+^CD45RO^+^CD45RA^-^ memory T cells compared to the control group by day 360 (21). They also demonstrated higher numbers of CD4^+^CD25^high^Foxp3^+^CD127^-^ Tregs than the control group (21).

Peng et al. used flow cytometry to monitor immune response post MSC treatment in renal transplantation (20). No difference was seen between treatment and control groups in numbers of CD4^+^ T cells, CD8^+^ T cells or NK cells before or after transplant (20). However, authors did note an increased proportion of CD27^+^ memory B cells in the MSC treated group (20). Casiraghi et al. reported a case report of tolerance following MSC infusion in liver transplant. They noted a high Treg:memory CD8^+^ T cells ratio, compared to control. They also demonstrated expansion in naïve and transitional B cells (26).

### Regulatory Dendritic Cells (DCs)

Macedo et al. used flow cytometry for immune monitoring post allogeneic DCreg infusion in living donor liver transplant patients. They reported a decrease in T-bet^+^Eomes^+^CD8^+^ T cells (both central and effector memory phenotypes) following DCreg infusion (24). Conversely there was an increase in T-bet^-^Eomes^-^CD8^+^T cells of naïve phenotypes with increased PD1^+^ and Tim3^+^ expression (24). Furthermore there was an increase in CD4^+^CD25^+^Foxp3^+^Tregs, resulting in a change to the Treg : CD8^+^ ratio and potentially a more tolerogenic profile (24).

## Functional Assays

Functional assays have an important part to play in immune monitoring. While flow cytometry is able to provide excellent data on the phenotype of cells, functional assays provide information on what role these cells may be playing. Functional assays include measurement of cell proliferation (as an indicator of activation; an example of *in vitro* suppression test with proliferation readout is shown in **Figure 2B**), measurement of chemokines or cytokines produced by cells, or their effect on other cells (for example lysis). The limitation to these studies is that the assays often require *ex vivo* manipulation of some description that may not reliably replicate what is happening *in vivo*. Below we review the functional assays that have been used to date in clinical trials of cellular therapy.

Mathew et al. used thymidine incorporation assays to test the immunocompetence of kidney transplant patients after Treg therapy (18). They looked at recipient PBMC response to a number of antigens and mitogens including PHA, ConA and CMV (18). Immediately after transplantation responses were low, likely due to induction immunosuppression, but they were shown to gradually recover in the first year post-transplant (18). Although they did not achieve pre-transplant levels it is useful to note that there were no clinical infections recorded in this time (18). Similarly, Casiraghi et al. used mixed lymphocyte reactions to demonstrate anti-donor CD8^+^ T cell unresponsiveness following MSC transfer in liver transplant recipients, suggestive of a more tolerant profile (26).

As well as providing analysis of peripheral blood leukocyte subsets, flow cytometry may be used to look at the proliferation profile of cells. Mudrabettu et al. labeled peripheral blood mononuclear cells (PBMCs) with carboxyfluoroscein succinimidyl ester (CFSE) prior to stimulating them with anti-CD3, anti-CD28 and IL-2. The proliferation profile of CD4^+^ T cells could then be measured using flow cytometry on the basis of CFSE staining (27). They demonstrated an initial decrease in proliferation in both control and treatment groups, likely secondary to immunosuppression. However, by day 90 after infusion the MSC treated group had a decrease in proliferation compared to control (27). Peng et al. used the same method but found no significant differences between control and MSC-treated groups (20).

The Pleximmune™ test (Plexision Inc, USA) is another functional assay that uses flow cytometry to quantify recipient CD8^+^CD45RO^+^ memory T cells expressing CD154 after they have been cultured for 16 hours with surrogate donor PBMCs. It was used by Sánchez-Fueyo et al. to establish the donor specific alloimmune response and demonstrated hyporesponsiveness in those who had received Treg infusion (25), suggesting movement towards a more tolerogenic phenotype. It is also important to check the function of the immune cells that are being transferred to the patients. For example, CD137^+^/CD154^-^ Tregs have been shown to be reliably associated with a stable phenotype (28). Upon a short stimulation with a relevant antigen, antigen-responsive Tregs express CD137, while antigen-responsive conventional CD4 T cells express CD154 (please see an example in **Figure 2C**). The CD137/CD154 assay has been used in the ONE Study to monitor the frequency of donor-responsive Tregs and conventional CD4 T cells.

Many studies have measured cytokine and chemokine levels in patient serum. Sanchez-Fueyo et al. used LEGENDplex™ (BioLegend) to assess cytokine and chemokine (including IL-2, -5, -12, -27 and CXCL9 and 10) levels post infusion. In one patient who concurrently had a high fever these levels were raised, but in all other patients no significant changes were found. The LEGENDplex™ is a bead-based immunoassay that captures the soluble analyte between two antibodies, before then quantifying the amount using flow cytometry (25). Roemhild et al. utilized Luminex (another bead-based immunoassay) for assessment after autologous polyclonal Treg therapy in renal transplantation, as part of the ONE study (23), there was no change in either pro- or anti-inflammatory cytokines (TNFα, IFNγ, IL-1, -6, -8 or-10. Shi et al. used ELISA to measure TGFß1 and PGE2, soluble factors released by MSCs that can modulate T and B cells. Both were increased at 4 weeks following MSC infusion (29). The Neptune study measured a number of cytokines and chemokines both before and 4 hours after MSC infusion (22). Using Biorad multiplexed assays they found that TNFα and IL-10 were both decreased following the second infusion of MSCs, a result that was maintained for the rest of the study. They did not find any significant differences in IL-4 or IFNγ (22).

Perico et al. monitored the alloimmune response to donor and third party antigen by using ELISpot assays to IFNγ, granzyme B and by cell-mediated lympholysis (21). They found that patients treated with autologous MSCs had decreased anti-donor IFNγ memory T cell and anti-donor granzyme B CD8^+^ cell responses compared to the control group (21). They also demonstrated a decreased cytolytic response of CD8^+^ T cells (21). Similarly, ELISpot assays have been used in the ONE Study and reported by Sawitzki et al, Roemhild et al. and Harden et al. (16, 23, 30).

The Cylex Immuknow Assay is used to test the immune competence of a patient’s T cells, by measuring the ATP synthesis of CD4^+^ T cells. Todo et al. used this as part of their immune monitoring of patients who received Treg therapy after liver transplantation (19). They were able to demonstrate results in the normal range for the majority of their participants (19).

The majority of these studies also tested for the presence of donor specific antibodies (DSAs), in particular development of *de novo* DSAs after transplantation (16, 18, 19, 22–24). There was not a significant increase in patients developing dnDSAs in these preliminary trials. This was the primary method for monitoring the humoral response. As discussed in section 2 (flow cytometry) immune monitoring also frequently included panels specifically to look at the B cell subsets over time post transplantation.

It would be interesting to know how the results of this immune monitoring correlate to clinical outcomes, however the studies discussed in these sections have all been early case reports or phase I trials, therefore focused on safety data and dose optimization.

## Next-Generation Technologies

### CyTOF

Mass spectrometry with cytometry by time of flight (CyTOF) is a key technology in multiple clinical trials where deep cellular phenotyping is important (31). In traditional flow cytometry, detection of fluorochrome-conjugated antibodies is based on wavelength in which sufficiently broad emission bands are produced. In mass cytometry, fluorescent labels are replaced with heavy metal tags that produce more narrow emission bands, as detection is based on mass. This limits signal overlap of emission spectra and facilitates an increased number of parameters to be simultaneously measured. Furthermore, these metals are not commonly found in biological specimens, reducing potential background noise (e.g. from autofluorescence) (31, 32).

CyTOF has the potential to overtake flow cytometry as the method of choice for immune monitoring (example of CyTOF data is shown in **Figure 2B** **)**. It is possible to stain intracellularly, therefore gaining functional insights and to look at antigen specificity of T cells by using metal conjugated tetramers, as well as allowing a high number of cell surface markers to be concomitantly identified (31). Furthermore, analysis is unbiased, with the potential to uncover new insights into immune cell subsets. There are some limitations, it is a slow and currently expensive method in comparison to flow cytometry and cells are not available for further studies at the end of the workflow.

Sánchez-Fueyo et al. used CyTOF to characterize the Treg compartment in patients following autologous Treg infusion. They were able to identify the expanded Tregs by comparing phenotypes of individual clusters to those examined pretransfusion. They could then follow them over time, noting by one month they had mostly disappeared. The expanded Tregs were found to be more proliferative and have increased CD25, CTLA4, CD38, GATA3, PD1 and CD274 (25).

Similarly, data from a cohort of patients enrolled in the ONE Study examined how the phenotype of Tregs changed over time post transplantation using CyTOF. Distinctive alterations were observed in clustering associated with specific post-transplantation changes. There were significant changes in the frequency of homing markers and CCR7^+^ Tregs (30).

### Gene Expression Profiling

Within transplantation research the focus of gene expression profiling has been to identify biomarkers of rejection or tolerance. A variety of techniques, including microarray and RT-qPCR, have been used to explore the potential mechanisms involved. However, at present gene expression profiling has not widely been used as part of the immune monitoring strategy post cellular therapy.

The potential importance of gene expression profiling in immunomonitoring has been recognized by the Banff Foundation, who created a molecular diagnostics working group to assess the available literature on this topic and plan for future research. At the latest symposium in 2019 they reached a consensus on a panel of 770 genes, to cover the innate and adaptive immune response, tolerance, rejection and infection for the monitoring of transplant patients, the Banff – Human Organ Transplant (B-HOT) panel (33). In collaboration with NanoString Technologies, this has become a commercially available panel. Of note it is possible to use with formalin fixed paraffin embedded (FFPE) samples, enabling retrospective analysis of stored samples (schematic overview of the method and an example of gene expression profiling using the B-HOT panel are shown in **Figures 3A, B** **)**.

As previously highlighted, the goal of cellular therapy is to reduce the need for harmful immunosuppression by inducing a tolerogenic state in patients. Therefore, when monitoring these patients it may be beneficial to use already developed gene signatures of tolerance as a standard for comparison, such as that described by Sagoo et al. (34). Indeed this was utilized by Hutchinson et al. to monitor patients being treated with Mreg therapy (35). They used microarray platforms to compare gene expression in their patients to those of a known tolerant cohort, finding them to be similar (35). Furthermore they found that TOAG mRNA expression (known to be decreased in acute rejection) remained high in treated patients, supporting a phenotype more often seen in healthy or tolerant patients (35).

As these biomarker profiles of tolerance or rejection become validated, it is possible to imagine how they may be used as a control or comparator group for those patients who have undergone cell therapy, to gain further insights into how these therapies are affecting the immune response.

### Spatial Biology

In recent years there have been significant developments in spatial profiling techniques, making this technology Nature’s 2020 Method of the Year (36). In general terms these offer the possibility of extracting spatially-resolved molecular information from tissue biopsies. Whilst bulk sequencing techniques generate detailed readouts of gene expression, they potentially miss out on small differences on a cell-per-cell basis that may be significant when taken in context of the position in which they occur. These newer techniques offer the possibility to perform more in-depth, spatially guided molecular analyses of tissue biopsies, which may be particularly relevant when trying to understand the effect of cellular therapies. We will briefly consider a few of the available techniques below.

#### Nanostring GeoMx DSP

The GeoMx DSP enables spatially-resolved, high-plex (10s -10,000s) digital quantitation of proteins and mRNA in tissue. It uses photo-cleavable oligo-tags to collect samples in a non-destructive way, whilst maintaining spatial information (37). Benefits include the direct, digital counting of mRNA or protein without the need for intervening enzyme steps, the ability to use archival FFPE samples, and the advantage that samples can be used for further downstream processing even after running through the GeoMx DSP workflow (schematic overview of the method and an example of kidney biopsy analyzed using GeoMx DSP are shown in **Figures 3C, D**).

#### 10X Genomics

This uses positional molecular barcodes in the cDNA synthesis reaction with an intact tissue section, before proceeding to generating a readout *via* RNA-seq (38). It offers the same highly spatially resolved readout as the GeoMx DSP but at present is only available for use with fresh frozen samples.

#### Fluidigm Hyperion

This platform works in a similar fashion to the GeoMx, but instead of oligo-tags it uses metal-conjugated antibodies, followed by laser ablation and transfer of the ablated tissue to be measured by CyTOF. It can be used with FFPE samples and can look at up to 35 different antigens at one time (39).

#### GE Cell Dive

This may also be used with FFPE samples or tissue microarray. After staining with dye conjugated biomarker antibodies (up to four) and collecting an image, it then uses a patented dye inactivation process to allow further staining of the same sample with different antibodies (up to 60 in total). These images are stitched together for a highly multiplexed final result (40).

#### Codex

This platform uses immunofluorescence technology, with an iterative workflow that uses DNA tagging technology (with capture and reporters). One of the benefits of this technology is the ability to comprehensively image the whole sample and perform unbiased cell phenotyping, rather than needed to choose regions of interest at the beginning (41).

### Cell Tracking

An excellent review by Tran and Thomson covers the current state of research into the tracking of adoptively transferred cells (8). This is a significant gap in our knowledge of the mechanism of action of these cellular therapies and may provide insight into how they modulate the immune response. Ashmore-Harris et al. have reviewed in detail the principles of non-invasive cell tracking, the methods available and how they may be used to develop new cellular therapeutics (42).

Hutchinson et al. pioneered the use of Mreg therapy in two kidney transplant patients (35). A proportion of the transfused Mregs were labeled with 45 Mbq oxine, allowing them to track where the cells went (using SPECT). They noted that after initially settling in the lungs they went on to travel in the circulation to liver, spleen and bone marrow. None were seen in the urinary tract, suggested good survival.

Chandran et al. monitored the fate of polyclonal Treg infusion in kidney transplant patients by labeling a proportion of transferred Tregs with deuterium and then monitoring levels over time. They found a peak in number in the first week, with the labeled Tregs still present at 30days. Numbers had fallen below the limit of detection by 3 months (43). This technique was developed by Bluestone et al. to monitor the fate of transferred polyclonal Tregs in a phase I trial in patients with type 1 diabetes (44). They also noted maximum levels of labeled Tregs at 7-14 days. Following which deuterium-Tregs decreased, to 25% of circulating Tregs at 90 days. Levels then stabilized over the next nine months. Furthermore, T effector populations did not demonstrate evidence of deuterium labeling, suggesting the transferred Tregs were stable in their identity (44).

## Discussion

In this paper we have reviewed the current immunomonitoring strategies used in the early and ongoing clinical trials of cellular therapy in transplantation as well as considering methods that may be of use in the future. At present the cornerstone of monitoring relies on flow cytometric analysis of peripheral blood samples to define the leukocyte subsets present. Standardized panels have been developed to enable comparison across clinical sites with good effect. These are often used in conjunction with an array of functional studies.

It is clear that the timing of sample acquisition is an important factor in any monitoring strategy. It is known that both induction and maintenance immunosuppression may have an effect on the immune cell subsets present. Indeed, a significant initial decrease in leukocytes, with slow recovery of T cell populations was demonstrated in a number of the studies discussed in this paper. This should be accounted for both when planning trials and reporting on the results. The ONE Study had a clear protocol for when samples were taken, together with the same immunosuppression, allowing for a harmonized analysis across groups.

There are a growing number of new techniques that offer the potential to explore both the phenotype and function of the immune response to cellular therapies. In particular, we are now in an era of deep spatial profiling which allows us to directly analyze transplant biopsies – the principal sites of activity - rather than surrogate tissues such as blood. These techniques offer the exciting possibility of discovering new tissue-specific treatment targets. However, the challenge will be in the analysis of data generated from these studies, which can be vast and open to misrepresentation. It will therefore be important to develop transparent standardized bioinformatic workflows to support the analysis and cross-site comparison of these data in order to fully understand their implications.

## Author Contributions

All authors contributed to the content design, literature searches, writing of the manuscript, and manuscript review. All authors contributed to the article and approved the submitted version.

## Funding

The work from authors’ own laboratory described in this manuscript have been supported by grants from Medical Research Council (MR/N027930/1), European Union Framework 7 programme though the ONE Study consortium, the Wellcome Trust (211122/Z/18/Z) and Kidney Research UK (SF1/2014). Additionally, the authors acknowledge funding from the EU Horizon 2020 Research and Innovation Programme under grant agreement 825392 (RESHAPE). HS is funded by a Royal College of Surgeons Research Fellowship. AA is funded by King Abdulaziz University, Saudi Arabia. JH is a Kidney Research UK Senior Research Fellow. FI is a Wellcome Trust CRCD Fellow.

## Conflict of Interest

The authors declare that the research was conducted in the absence of any commercial or financial relationships that could be construed as a potential conflict of interest.
